# Biochemical characterization of P-type copper ATPases

**DOI:** 10.1042/BJ20140741

**Published:** 2014-09-22

**Authors:** Giuseppe Inesi, Rajendra Pilankatta, Francesco Tadini-Buoninsegni

**Affiliations:** *California Pacific Medical Center Research Institute, San Francisco, CA 94920, U.S.A.; †Department of Biochemistry and Molecular Biology, Central University of Kerala, Padennakkad (P.O), Nileshwar, Kasaragod-671 314, Kerala, India; ‡Department of Chemistry “Ugo Schiff”, University of Florence, 50019 Sesto Fiorentino, Italy

**Keywords:** bacterial copper ATPase, cellular trafficking, copper displacement, cisplatin, mammalian copper ATPase interaction, serine phosphorylation, BCS, bathocuproine disulfonate, NMBD, N-terminal metal-binding domain, PKD, protein kinase D, TMBS, transmembrane metal-binding sites, WT, wild-type

## Abstract

Copper ATPases, in analogy with other members of the P-ATPase superfamily, contain a catalytic headpiece including an aspartate residue reacting with ATP to form a phosphoenzyme intermediate, and transmembrane helices containing cation-binding sites [TMBS (transmembrane metal-binding sites)] for catalytic activation and cation translocation. Following phosphoenzyme formation by utilization of ATP, bound copper undergoes displacement from the TMBS to the lumenal membrane surface, with no H^+^ exchange. Although PII-type ATPases sustain active transport of alkali/alkali-earth ions (i.e. Na^+^, Ca^2+^) against electrochemical gradients across defined membranes, PIB-type ATPases transfer transition metal ions (i.e. Cu^+^) from delivery to acceptor proteins and, prominently in mammalian cells, undergo trafficking from/to various membrane compartments. A specific component of copper ATPases is the NMBD (N-terminal metal-binding domain), containing up to six copper-binding sites in mammalian (ATP7A and ATP7B) enzymes. Copper occupancy of NMBD sites and interaction with the ATPase headpiece are required for catalytic activation. Furthermore, in the presence of copper, the NMBD allows interaction with protein kinase D, yielding phosphorylation of serine residues, ATP7B trafficking and protection from proteasome degradation. A specific feature of ATP7A is glycosylation and stabilization on plasma membranes. Cisplatin, a platinum-containing anti-cancer drug, binds to copper sites of ATP7A and ATP7B, and undergoes vectorial displacement in analogy with copper.

## INTRODUCTION

**Scheme 1:**
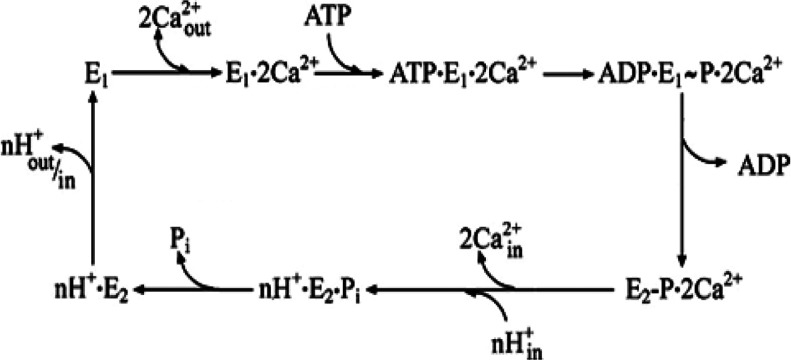
Schematic diagram of sequential reactions in the catalytic cycle of the Ca^2+^ ATPase Following Ca^2+^ binding to the enzyme in state E1, ATP utilization yields a phosphorylated intermediate, whereupon utilization of phosphorylation potential yields the E2 conformation, which allows vectorial displacement and release of bound Ca^2+^ in exchange for H^+^.

The superfamily of P-type ATPases comprises membrane-bound enzymes that utilize the potential energy of ATP for active transport of ions [1–4]. The P-type denomination refers to (alkali-labile and acid-stable) phosphorylation of an aspartate residue by ATP within the catalytic site. This phosphorylation reaction is an intermediate step for energy transduction and vectorial displacement of bound ions, before hydrolytic cleavage of P_i_. The structure of these enzymes includes a headpiece above the membrane cytosolic surface, containing domains for ATP-binding (N domain), phosphoryl transfer (P domain) and catalytic activation (A domain). On the other hand, binding sites for specific ions [TMBS (transmembrane metal-binding sites)] reside within the membrane-bound region, with participation of residues from various transmembrane helices. Energy transduction occurs through a long-range mechanism, including sequential changes of protein conformation, allowing cation binding with high affinity on one side of the membrane, followed by vectorial displacement and release with lower affinity from the other side (Scheme 1).

Detailed characterization of P-type ATPases involved in active transport of Na^+^ or Ca^2+^ was favoured by their natural abundance in native membranes, permitting biochemical and crystallographic studies [5–11]. In addition, the concentrations of alkali and alkali-earth ions within membrane-delimited compartments are fairly high (0.1 μM-mM), permitting estimates of free ion concentrations and transmembrane electrochemical gradients, as well as determinations of bound ion stoichiometry by the use of radioactive tracers. On the other hand, characterization of transition metal ion P-ATPases has been more difficult due to low quantities of these enzymes in native membranes, and extremely low concentrations of transition metal ions (e.g. copper) within intra- and extra-cellular fluids. Furthermore, the physiology of cytosolic transition metal homoeostasis is rather complex, as it involves transporters such as chaperone proteins, delivering ions to P-ATPases on one side of the membrane, and receiving them from the other side. Important and general information on P-type copper ATPases and on related cellular mechanisms has been given previously [12–18]. The present review is focused on the biochemical properties of copper ATPases, reporting available information on catalytic events related to cation translocation, reactions favouring cellular trafficking of ATPases, and roles of various domains within the ATPase protein.

## BACTERIAL COPPER P-TYPE ATPases

Cu^+^-ATPases contribute to bacterial homoeostasis through their involvement in Cu^+^ detoxification and cuproprotein assembly. Original and fundamental contributions to characterization of bacterial copper ATPases were obtained by the use of the thermophilic *Archaeoglobus fulgidus* CopA as a model protein within the subfamily of PIB-type ATPases. Functional characterization [19] of the cloned and heterologously expressed protein indicated that this CopA enzyme is active at high temperature (75°C) in the presence of Ag^+^ or Cu^+^, utilizing ATP by formation of an acid stable intermediate, and proceeding with hydrolytic activity at a maximal rate of 3.66 μmol/mg per h in the presence of Cu^+^. The metal concentration dependence of enzyme activation observed is in the micromolar range (Supplementary Figure S1 at http://www.biochemj.org/bj/463/bj4630167add.htm).

**Figure 1:**
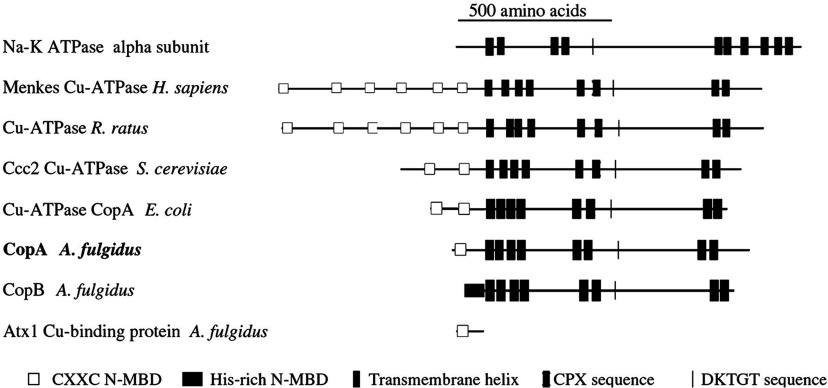
Membrane topology and sequential arrangement of conserved functional domains of CPx-ATPases Comparison among PIB-type ATPases from different species, with the Na^+^/K^+^-ATPase α-subunit (a typical PII-type ATPase) and a cytoplasmic copper chaperone. Reproduced with permission, from the American Society for Biochemistry and Molecular Biology; Copyright © 2002. This Figure was originally published in J. Biol. Chem. [19] Mandal, A.K., Cheung, W.D. and Argüello, J.M. Characterization of a thermophilic P-type Ag^+^/Cu^+^-ATPase from the extremophile *Archaeoglobus fulgidus*. *Journal of Biological Chemistry*. 2002; **277**, 7201–7208. © the American Society for Biochemistry and Molecular Biology.

A comparison (Figure 1) of PIB-type ATPases with the Na^+^/K^+^-ATPase α subunit (a typical PII-type ATPase) shows that the *A. fulgidus* CopA is an 804 amino acid protein matching the typical topological pattern of other PIB-type ATPases, with a large cytoplasmic loop containing the DKTGT sequence (as in the PII-type) and eight transmembrane segments (rather than ten as in the PII-type). Invariant residues in the transmembrane region (two cysteine residues and a tyrosine for site I, and an asparagine, a methionine and a serine for site II) contribute to two high-affinity TMBS, whose simultaneous occupancy is required for enzyme turnover [20,21]. Furthermore, the *A. fulgidus* CopA has an NMBD (N-terminal metal-binding domain) with a single Cu^+^-binding motif (CXXC) at the N-terminal, which is not present in PII-type ATPases. The NMBD increases in length, with a higher number of Cu^+^-binding motifs in other PIB-type ATPases, up to six in eukaryotic enzymes (Figure 1). Cryoelectron microscopy of bacterial CopA tubular crystals reveals that the NMBD interacts with the cytosolic headpiece, suggesting a Cu-dependent regulatory role for the NMBD [22].

With regard to protein structure, early crystallographic studies, utilizing isolated domains of *A. fulgidus* Cu^+^-ATPase, identified features of the headpiece domain, such as the actuator (‘A’) and ATP-binding (‘N’) domains, analogous to those of PII-type ATPase [23–25]. Additional information was obtained by 2D crystal electron cryomicroscopy of *Aquifex aeolicus* PIB-type ATPase, indicating the arrangement of transmembrane helices with respect to the enzyme headpiece [26]. Further progress was then made by Gourdon et al. [27] who obtained the crystal structure of the *Legionella pneumophila* CopA at 3.2 Å (1 Å=0.1 nm) resolution, in the copper-free state and in the presence of AlF_4_^−^, yielding a state analogous to E_2_**·**P_i_ (i.e. the transition state following hydrolytic cleavage of the phosphoenzyme intermediate in the final phase of the ATPase catalytic cycle, and preceding dissociation of inorganic phosphate from the enzyme).

**Figure 2:**
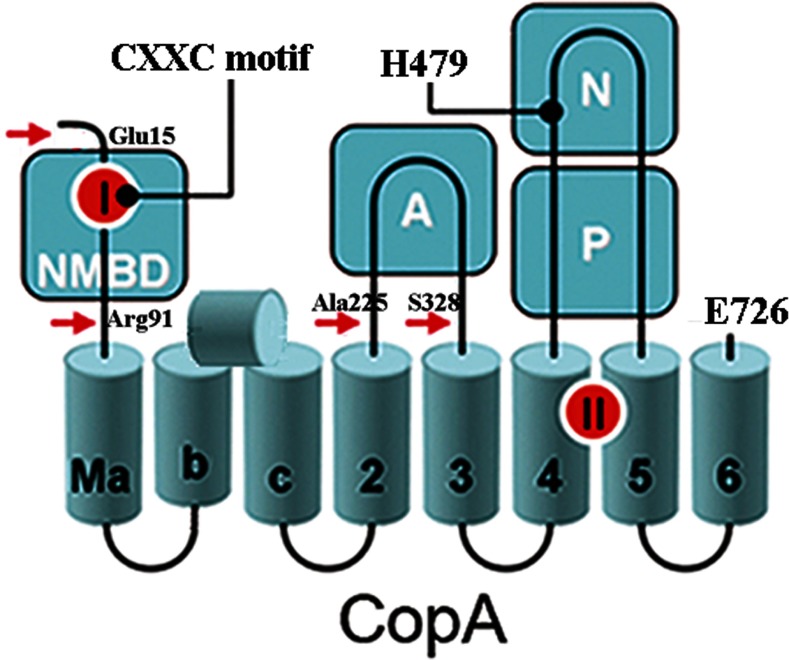
Folding diagram of the (bacterial) *T. maritima* CopA sequence The full-length sequence comprises 726 amino acids, including an NMBD, followed by four closely spaced transmembrane segments (Ma, Mb, Mc and M2) and an extramembranous A domain. The sequence continues with two (M3 and M4) transmembrane segments, a large extramembranous region, including the N and P domains, and two closely spaced (M5 and M6) transmembrane segments at the C-terminus. A shorter MB, followed by an amphipathic segment on the cytosolic membrane surface is included in the diagram [27]. Two distinct copper-binding sites are coloured red on the NMBD and at the transmembrane site (TMBS). The headpiece includes the actuator domain (A) and the nucleotide-binding domain (N) interspaced within the phosphorylation domain (P) sequence where Asp^445^ undergoes phosphorylation. The locations (see text) of mutations in the NMBD (double mutation of C17A and C20A termed the CXXC mutant) and the N domain (H479Q), are indicated in the diagram. The fragments resulting from papain cleavage of the 14 N-terminal residues at the N-terminal end to Glu^15^, at the NMBD–Ma link (N-terminal end to Arg^91^), at the M2–A domain link (N-terminal end to Ala^225^), or at the A domain–M3 link (N-terminal end to Ser^328^) are also shown. The arrows point to papain cleavage sites. Adapted from [34] © 2009 American Chemical Society.

As suggested by sequence analysis, a schematic 2D diagram (Figure 2) of the high-resolution structure shows a headpiece with three domains (N, P and A) that are present in other P-type ATPases. There are, however, eight transmembrane segments (rather than ten as in PII-type ATPases, i.e. Ca^2+^ ATPase), with three N-terminal transmembrane segments considered specific and referred to as MA, MB and MC (or M1), followed by M2, M3, M4, M5 and M6 interfacing with the headpiece in analogy with PII-type ATPases. Furthermore, although in the PII-type ATPases the A domain interfaces with the first, second and third transmembrane segments, the A domain of the copper ATPase intervenes only between the M2 and M3 transmembrane segments, lacking interface with MC (M1 in PII-ATPase terminology). In addition, MB includes a kinked amphipathic helix placed on the cytosolic membrane surface. This PIB-specific transmembrane helix kinks at a double-glycine motif displaying an amphipathic helix that lines a putative copper entry point at the cytosolic interface. The MA transmembrane segment is preceded by the NMBD, including the CXXC motif, presumably for copper binding and enzyme catalytic regulation. On the other hand, six residues in the transmembrane domain (Cys^382^ and Cys^384^ in M4, Tyr^688^ and Asn^689^ in M5, Met^717^ and Ser^721^ in M6) overlap the location of Ca^2+^ co-ordinating residues in the Ca^2+^ ATPase (absent in the copper ATPase), and are postulated to be involved in binding of two copper ions at the TMBS, for enzyme activation and copper translocation following the formation of a phosphorylated enzyme intermediate.

Additional contributions to functional characterization of bacterial copper ATPases was obtained in experiments with recombinant and purified *Thermotoga maritima* CopA [28,29]. A schematic 2D diagram of the *T. maritima* CopA is shown in Figure 2, based on partial sequence homology with other cation transport ATPases and available structural information. It was found that this enzyme sustains an ATPase velocity of 1.78–2.73 μmol/mg per min in the presence of Cu^+^ (pH 6, 60°C), as compared with 0.03–0.08 μmol/mg per min in the absence of Cu^+^. It is noteworthy that different ATPase turnover rates have been reported for CopA enzymes derived from different organisms [30].

The Cu^+^ concentration dependence of *T. maritima* CopA ATPase activation is within the micromolar range (Supplementary Figure S2 at http://www.biochemj.org/bj/463/bj4630167add.htm). These measurements, performed with purified protein in order to characterize the biochemical properties of the enzyme, show that micromolar Cu^+^ can reach the activating ATPase sites directly. Note, however, that in the physiological cell environment free Cu^+^ is present at much lower concentrations, and Cu^+^ sequestered by chaperones is expected to be delivered directly to the ATPase TMBS through protein interactions, increasing its binding affinity to a very high level [31,32]. It is apparent that complementation of an electropositive platform in the ATPase and an electronegative Cu^+^ chaperone drives an interaction whereby Cu^+^ is placed proximal to conserved carboxy and thiol groups at the ATPase entrance site. Cu^+^ is then released from the chaperone via ligand exchange, and transferred on to the TMBS involved in transmembrane translocation [33]. At any rate, it is apparent that even in the absence of chaperones, Cu^+^ at the micromolar concentration level can reach the sites involved in ATPase activation, offering a convenient way to study the biochemical properties of the isolated enzyme.

**Scheme 2:**
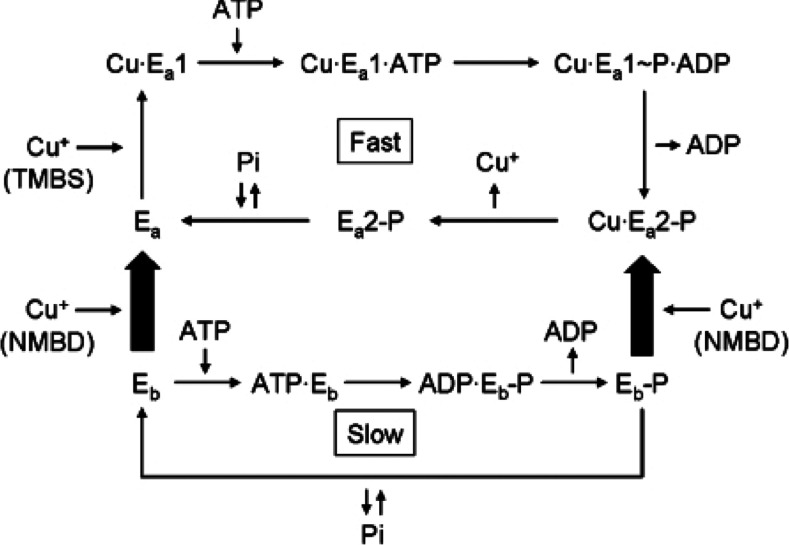
Diagram of the *T. maritima* CopA reaction sequences indicating that the enzyme can be phosphorylated by ATP, or even by P_i_ in the absence of Cu^+^, yielding a phosphoenzyme species (E_b_-P) undergoing very slow turnover The active enzyme conformation (E_a_) undergoing fast turnover is only obtained following Cu^+^ binding to both NMBD and TMBS. Reproduced from [34] © 2009 American Chemical Society.

Experiments with *T. maritima* CopA ATPase showed that deletion of the NMBD (ΔNMBD) or mutation of the NMBD Cu^+^-binding site (CXXC) resulted in negligible ATPase activity, even though high phosphoenzyme levels are obtained by utilization of ATP (or even P_i_) in the absence of Cu^+^ [29]. NMBD deletion or mutation experiments demonstrate that either in the absence of the segment or in the presence of the segment with no Cu^+^ bound, no catalytic turnover is observed. Therefore the presence of the NMBD segment with Cu^+^ bound is required to allow ATPase activation, as shown in Scheme 2.

**Figure 3:**
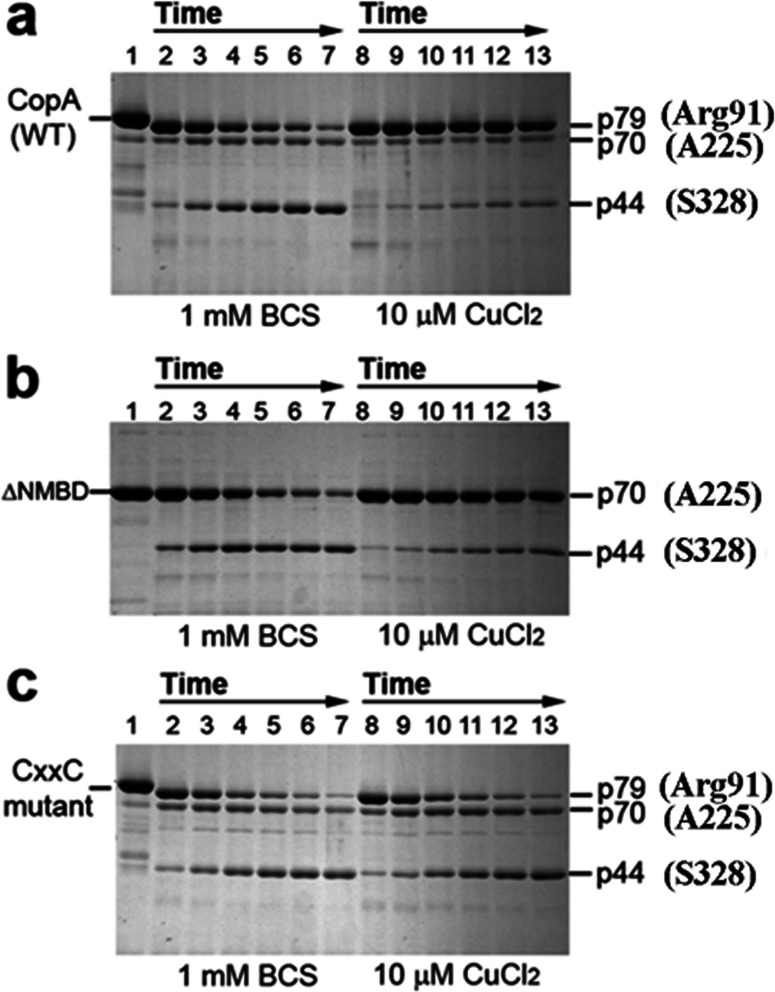
Effect of copper on the digestion pattern of *T. maritima* CopA WT, ΔNMBD (NMBD entirely removed) and CXXC (double mutation of C17A and C20A in the NMBD) proteins Purified proteins (0.5 mg/ml) of CopA WT (a) and the mutants (b and c) were incubated with 0.5 mg/ml papain at 50°C in the presence of 1 mM BCS (copper chelator) (lanes 2–7) or with 10 μM CuCl_2_ (lanes 8–13), as indicated at the bottom. The numbers on top of the gels refer to minutes of digestion: lane 1, 0 min; lanes 2 and 8, 2 min; lanes 3 and 9, 5 min; lanes 4 and 10, 10 min; lanes 5 and 11, 15 min; lanes 6 and 12, 20 min; and lanes 7 and 13, 30 min. The quenched samples were subjected to SDS electrophoresis. Reproduced from [34] © 2009 American Chemical Society.

It is unlikely that the NMBD site serves as a direct conduit for Cu^+^ transfer and delivery to the TMBS, since the variable NMBD stoichiometry (one in bacterial enzymes and up to six in mammalian enzymes) does not match the TMBS stoichiometry (i.e. two). Furthermore, it was clearly demonstrated that chaperone proteins are required to deliver Cu^+^ to the TMBS with high affinity, and such a high affinity cannot be achieved in the absence of chaperones even if the NMBD is present. It is then apparent that Cu^+^-NMBD is involved in conformational interactions with the A domain (see below), yielding ATPase activation by Cu^+^ bound to TMBS. Additional functions are sustained by the much longer NMBD segment of mammalian enzymes, as explained below.

Studies of the effects of ligands and mutations on the digestion pattern of CopA with papain demonstrate that protein conformational changes are of great importance in the regulation of catalytic kinetics [34]. Figure 3 shows that digestion of WT (wild-type) CopA with papain in the absence of copper [BCS (bathocuproine disulfonate) present] yields at first a p79 fragment, resulting from papain cleavage of the 14 N-terminal residues at the N-terminal end at Glu^15^, and a p70 fragment resulting from cleavage at the NMBD–Ma link at Arg^91^ (Figures 2 and 3). Then, a p44 fragment (Figure 3) is gradually produced through cleavage at the M2–A domain link at Ala^225^, and even smaller fragments following cleavage of the A domain–M3 link at Ser^328^. On the other hand, copper at concentrations permitting maximal ATPase activity (i.e. 10 μM) confers significant protection of the p79 and p70 fragments, whereby only small quantities of p70 and p44 are produced (Figure 3a). Thus copper reduces the susceptibility of both the NMBD–Ma loop (Arg^91^) and the A domain–M3 loop (Ser^328^) to digestion, suggesting that the NMBD segment and the A domain fold over and/or change their positions in the presence of Cu^+^.

If CopA with deletion of the NMBD (Figure 3: ΔNMBD) is used, the intact protein migrates as a p70 band reflecting the NMBD deletion. In the absence of copper, production of the p44 fragment (i.e. cleavage at Ser^328^ on the A domain–M3 loop) occurs within a time frame similar to that observed with the WT protein (compare digestion in panels a and b of Figure 3). In the presence of copper, the p70 protein is protected and the yield of p44 fragment is reduced, as observed with the WT protein. This indicates that the protective conformational effect is induced by copper binding to the TMBS, since the NMBD is absent. Therefore Cu^+^ can be bound by TMBS even in the absence of the NMBD segment.

If the NMBD CXXC mutant is used, the same digestion pattern observed with both WT and ΔNMBD in the absence of copper is obtained. However, in this case, addition of copper does not produce any protection (Figure 3c), indicating that the copper-free NMBD (CXXC mutant) prevents the protective conformational change induced by the copper binding to the TMBS, as required for enzyme activation.

The experimental evidence reported so far indicates that, following catalytic activation by concerted effects of NMBD and TMBS copper occupancy, ATP is utilized in analogy to other P-type ATPases to form [2Cu^+^·E1 P] where E1 P is a high-energy phosphorylated intermediate with 2Cu^+^ bound at the TMBS sites, expected to undergo vectorial translocation (Scheme 1). The phosphorylated moiety potential energy is then utilized for the conformational transition of [2Cu^+^·E1 P·P] to [2Cu^+^·E2-P], thereby reducing the affinity and changing the orientation of Cu^+^ bound to the TMBS sites. Vectorial displacement of bound Cu^+^ would then be followed by hydrolytic cleavage of P_i_, yielding E2·P_i_ and final release of P_i_. It is apparent that interaction of NMBD with the catalytic headpiece, and consequent A domain movements, play important roles in the sequential reactions of the ATPase cycle. This may be accomplished by facilitating Cu^+^ entry to the TMBS and, most importantly, by allowing proper placement of the A domain conserved catalytic sequence near the P domain phosphorylated site, thereby facilitating the hydrolytic reaction. Copper occupancy of the NMBD remains most likely throughout the cycle, whereas copper bound to the TMBS is released with each cycle.

It is of interest that defective ATP utilization is produced by a histidine residue mutation (H479Q) in the bacterial *T. maritima* CopA (Figure 2), in analogy to that produced by the corresponding mutation in human Wilson disease copper ATPase (ATP7B) [34].

Specific and novel information on the final steps of the *L. pneumophila* CopA catalytic cycle was obtained following crystallization of the enzyme in the absence of copper and in the presence of BeF^3−^, yielding an E2-P state analogue. It was inferred from this crystal structure that the enzyme transmembrane domain positions in E2-P and E2·Pi states retains structural similarity [35]. In addition, molecular dynamics simulations show extracellular water solvated within the transmembrane domain, indicating that the transmembrane domains of the E2-P and the E2·Pi states are both open towards the extracellular side, thus suggesting a class-specific ion-release pathway originating from ion-binding residues deep within the transmembrane domain, allowing Cu^+^ release as shown in Scheme 2 (deeper colour).

**Scheme 3:**
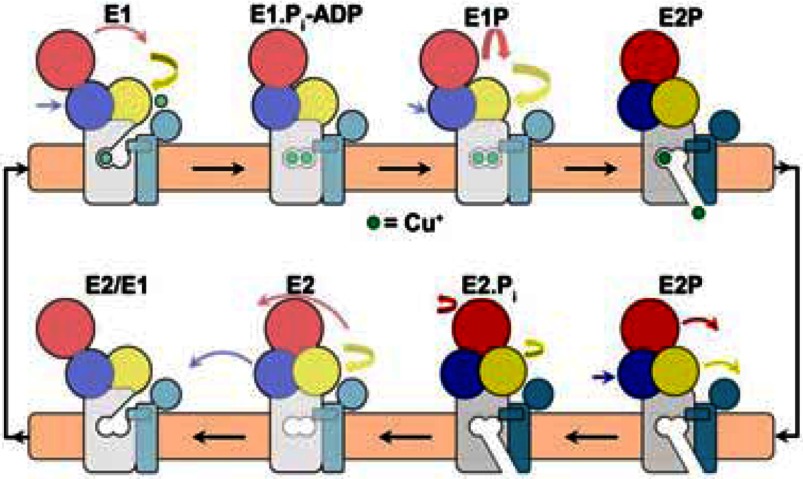
Reaction scheme proposed for the *L. pneumophila* CopA, where the headpiece domains are shown in yellow (A), red (N), blue (P) and light blue (NMBD), and the coloured arrows indicate their movements Note that the transmembrane domains undergo no major movements in the transition between E2-P and E2**^.^**P_i_. However, as explained above, conformational transitions occur upon Cu^+^ binding to the NMBD and TNBS, as well as upon utilization of ATP. Most importantly, the (Cu^•^) E1 P to (Cu^•^) E2-P conformational transition is related to utilization of phosphorylation potential, resulting in vectorial displacement of bound Cu^+^. Reprinted by permission from Macmillan Publishers Ltd: *Nature Structural and Molecular Biology* [35] Andersson, M., Mattle, D., Sitsel, O., Klymchuk, T., Nielsen, A. M., Møller, L. B., White, S. H., Nissen, P. and Gourdon, P. (2014) Copper-transporting P-type ATPases use a unique ion-release pathway. Nat. Struct. Mol. Biol. **21**, 43–48, copyright (2014)

It should be pointed out that, in contrast with CopA (Scheme 3), a conformational change between E2-P and E2·Pi was clearly demonstrated for the Ca^2+^ ATPase [36], consisting of movements and rotation of transmembrane helices, opening and closing a switch of the lumenal Ca^2+^ release gate, in conjunction with H^+^ exchange. Therefore the crystal structures of Cu^+^-ATPase from *L. pneumophila* obtained so far, in conjunction with site-directed mutagenesis and molecular dynamics simulations, indicate a copper transport pathway across the membrane, where Cu^+^-ATPases couple dephosphorylation and ion extrusion differently than do the well-characterized PII-type ATPases [37]. Furthermore, although Ca^2+^/H^+^ exchange occurs in the Ca^2+^ ATPase (Scheme 1), no evidence of counterion binding is available for the Cu^+^-ATPase.

## MAMMALIAN COPPER P-TYPE ATPases

The identity and importance of human P-type copper ATPases was first noted in genetic analysis of patients affected by Menkes’ disease, demonstrating abnormalities in a gene related to P-type cation-transport ATPases [38]. This enzyme was then named ATP7A, and will be referred to as such in the present review. It was also reported that the gene responsible for Wilson's disease encodes a copper transport ATPase that has homology with the Menkes’ disease gene [39–41]. This enzyme was then named ATP7B, and will be referred to as such in the present review. Subsequent studies, reviewed by Lutsenko et al. [42] and Veldhuis et al. [43], expanded information on the structural organization and functional properties of the ATP7A and ATP7B isoforms. It is clear that the two enzymes play an essential role in cellular physiology, as they are involved in transfer of dietary copper from enterocytes into blood, export of copper from liver into bile, maintain intracellular copper below toxic levels by translocation from the cytosol across cellular membranes, and contribute to protein biosynthesis by delivering copper into the lumen of the secretory pathway where the metal ion is incorporated into copper-dependent enzymes. They also sustain specialized roles such as systemic copper absorption (ATP7A) and copper excretion (ATP7B), and maintain copper homoeostasis in various tissues such as brain, kidney, lungs, mammary gland, heart and placenta. Important properties of these enzymes are related to their trafficking [16,44] between the *trans*-Golgi network and exocytic vesicles located at or proximal to the apical (ATP7B) or basolateral (ATP7A) cell surface. Interaction with other proteins and involvement of signalling kinases appear to be involved in the activating function and trafficking of mammalian copper ATPases.

**Figure 4:**
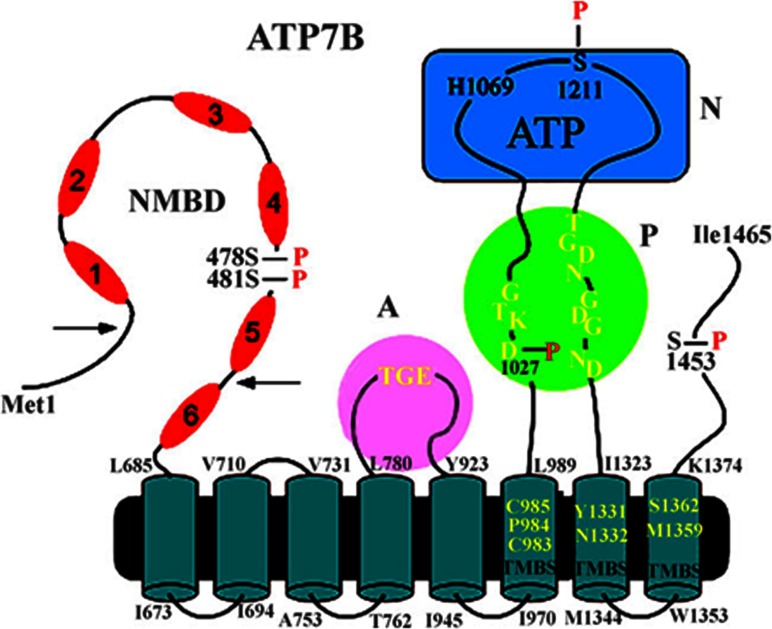
2D folding model of the (mammalian) ATP7B sequence The diagram shows eight transmembrane segments including a copper-binding site (TMBS). Yellow residues in the TMBS are probably involved in copper binding. The extramembranous region comprises a nucleotide-binding domain (N) with the His^1069^ residue whose mutation is frequently found in Wilson's disease; the P domain, with several residues (in yellow) conserved in P-type ATPases, where Asp^1027^ undergoes phosphorylation to form the catalytic phosphoenzyme intermediate (EP); the A domain with the TGE (yellow) conserved sequence involved in catalytic assistance of EP hydrolytic cleavage; the NMBD with six copper-binding sites; and a C-terminal chain. As suggested in [46], a kinked amphipathic segment of the second helix may overlap the cytosolic membrane surface. Serine residues undergoing kinase-assisted phosphorylation (Ser^478^, Ser^481^, Ser^1121^ and Ser^1453^) reside within flexible loops of the protein. This Figure was adapted from J. Biol. Chem. [55] Pilankatta, R., Lewis, D., Adams, C.M. and Inesi, G. C. High yield heterologous expression of wild-type and mutant Cu^+^-ATPase (ATP7B, Wilson disease protein) for functional characterization of catalytic activity and serine residues undergoing copper-dependent phosphorylation. *Journal of Biological Chemistry*. 2009; **284**, 21307–21316. © the American Society for Biochemistry and Molecular Biology and J. Biol. Chem [60] Pilankatta, R., Lewis, D. and Inesi G. Involvement of protein kinase D in expression and trafficking of ATP7B (copper ATPase). *Journal of Biological Chemistry*. 2011; **286**, 7389–7396

The structure of mammalian copper ATPases (Figure 4), in analogy with bacterial copper ATPases [45], comprises a headpiece with the N, P and A domains and conserved catalytic motifs, and eight transmembrane segments including TM4, TM5 and TM6 that display characteristic CPX motifs for copper binding (TMBS) involved in catalytic activation and subjected to vectorial translocation. A specific feature of mammalian copper ATPases is an NMBD comprising six (rather than one as in the bacterial enzyme) copper-binding sites [46–49]. The NMBD extension, shown to comprise flexible elements [50], is involved in interactions with partner proteins and sustains copper-induced and functionally relevant conformational effects. Although the crystal structure of the mammalian copper ATPases is not available as yet, solution structures of specific domains have been obtained by spectroscopic methods [50–52].

### Phosphoenzyme formation and vectorial displacement of bound copper

The native abundance of copper ATPases is quite low and, for this reason, biochemical experimentation has been conducted with recombinant protein obtained by heterologous expression in yeast or insect cells [48,53,54], providing evidence for phosphorylation upon addition of ATP. Characterization of the biochemical properties of mammalian P-type copper ATPases were significantly aided by high-yield heterologous expression in cultured mammalian cells following cDNA transfer with viral vectors [55]. It was found that the microsomal fraction of cell homogenates containing heterologously expressed ATP7B sustains a copper-dependent steady-state ATPase activity of 50–150 nmol of P_i_/mg of protein per min at 37°C, pH 6.0. Since the expressed ATP7B accounts for approximately 20% of the total protein, it can be estimated that the specific ATPase activity would be 0.25–0.75 μmol/mg of ATP7B protein per min. This value is much lower than that observed with the Ca^2+^ ATPase SERCA1 (sarcoplasmic/endoplasmic reticulum Ca^2+^-ATPase 1) under optimal conditions (2–5 μmol/mg per min). The lower rate was confirmed by measurements of exponential phosphoenzyme decay (see Supplementary Figure S5) after its formation by ATP addition to enzyme pre-equilibrated with Cu^+^, under conditions which are not influenced by the enzyme concentration and may not be influenced by chaperones affecting copper delivery before phosphoenzyme formation. The phosphorylation experiments yield basic information on kinetic features of the ATPase, such as phosphorylated intermediate formation and decay, as their rates are influenced by specific interactions (i.e. NMBD), site-directed mutations and deletions. This biochemical information may become very useful to interpret crystallographic data when available.

Another important feature permitted by the high-yield expression is the demonstration that, upon addition of ATP, enzyme phosphorylation occurs with biphasic kinetics, and steady-state hydrolytic cleavage of P_i_ begins in conjunction with the fast component of alkali labile phosphoenzyme production, and does not increase further as protein phosphorylation continues at a slow rate to produce alkali-resistant phosphoprotein (Supplementary Figure S3 at http://www.biochemj.org/bj/463/bj4630167add.htm). Only the first component corresponds to phosphorylation of Asp^1027^ (P domain) to form the phosphoenzyme catalytic intermediate which, as expected, is stable at acid pH and labile at alkaline pH. In fact, its formation is totally prevented by site-directed D1027N mutation (Supplementary Figure S4 at http://www.biochemj.org/bj/463/bj4630167add.htm). In addition, exponential decay of the acid-stable fast component of [^32^P]phosphoenzyme (obtained with [γ-^32^P]ATP) is observed following chase with non-radioactive ATP [56], and occurs with a rather slow decay rate (Supplementary Figure S5 at http://www.biochemj.org/bj/463/bj4630167add.htm), consistent with the steady-state ATPase velocity, and corresponds to the rate constant of phosphoezyme intermediate hydrolytic cleavage in the forward direction of the catalytic cycle [56].

It has become possible, using a technique based on absorption of membrane-bound ATPase on to a hybrid alkane thiol/phospholipids bilayer anchored to a gold electrode (solid supported membrane), to detect charge movements due to ion displacement within the ATPase protein upon addition of ATP [57]. The electrical current recorded by this method is a measure of the rate of change of the transmembrane potential and is not sensitive to steady-state phenomena. Therefore only the charge movement within the first enzyme cycle is recorded. Using microsomes of COS1 cells expressing ATP7A or ATP7B with high yield, it was then demonstrated with pre-steady-state current measurements that within a single catalytic cycle following addition of ATP, TMBS-bound copper undergoes electrogenic movement within the ATP7B protein, consistent with vectorial displacement towards the lumenal membrane surface [58]. This charge movement is totally prevented by mutations of either the TMBS site or the sixth NMBD copper-binding site, demonstrating the specificity of this phenomenon with regard to the enzyme cycle. Comparison of the time constants for cation displacement in the copper and in the calcium transport ATPases [58] is consistent with the slower copper ATPase phosphoenzyme formation. Interestingly, although charge transfer is reduced by alkaline pH in the Ca^2+^ ATPase (Supplementary Figure S6 at http://www.biochemj.org/bj/463/bj4630167add.htm) since Ca^2+^/H^+^ exchange is required for vectorial displacement of bound Ca^2+^ [59], no effect of pH is observed with the copper ATPase (Supplementary Figure S6) indicating that copper displacement does not require H^+^ exchange [56]. It is clear that the initial formation of alkali-labile phosphoenzyme upon addition of ATP is soon followed by vectorial displacement of bound copper from the TMBS to the lumenal membrane surface.

### Phosphorylation of serine residues, the NMBD segment and ATP7B trafficking

The slower component of ATP7B phosphorylation by ATP occurs with the same pattern *ex vivo* (cell cultures) and *in vitro* (microsomes). Using microsomes of COS1 cells expressing ATP7A or ATP7B with high yield, it was then possible to demonstrate [55] by proteolysis and MS that slow phosphorylation involves Ser^478^ and Ser^481^ (NMBD), Ser^1121^ (N-terminal domain) and Ser^1453^ (C-terminus), as shown in Figure 4. Most importantly, although formation of enzyme intermediate is due to direct phosphorylation of Asp^1027^ by ATP, phosphorylation of serine residues is catalysed by PKD (protein kinase D), and is abolished by specific PKD inhibition with CID755673 [60]. The same pattern of phosphorylation is observed with ATP7B expressed in COS1 cells or hepatocytes. Under comparative conditions, it is found that the slow alkali-resistant phosphorylation does not occur at all with the Ca^2+^ ATPase (Supplementary Figure S4).

Experiments conducted following site-directed mutations demonstrate that aspartate residue (Asp^1027^) phosphorylation is interfered with by either mutations involving the TMBS or the sixth NMBD copper site (Supplementary Figure S7 at http://www.biochemj.org/bj/463/bj4630167add.htm), whereas inhibition of serine phosphorylation is only observed following mutation of the sixth NMBD copper site. It is of interest that, following deletion of the terminal NMBD segment including five copper sites (Δ5 protein), aspartate phosphorylation (but not serine phosphorylation) still occurs [56] as long as copper is bound to the TMBS (Supplementary Figure S8 at http://www.biochemj.org/bj/463/bj4630167add.htm). Such an intermediate undergoes the expected catalytic decay. This suggests that, in the absence of copper, the NMBD interacts with the enzyme headpiece, interfering with formation of the phos-phorylated enzyme intermediate (i.e. aspartate) even in the presence of copper on the TMBS. This interference can then be removed by copper binding to the NMBD, consistent with previous observations by Huster and Lutsenko [49].

Even more intriguing results are obtained when ATP7B and Δ5 (deletion of the first five NMBD sites) proteins are tested for their ability to undergo reverse phosphorylation by P_i_, a reaction obtained with the Ca^2+^ ATPase as long as Ca^2+^ is removed to destabilize the protein. It was then found that WT ATP7B is unable to react with P_i_ even if copper is removed. On the other hand, the Δ5 protein reacts with P_i_ [56]. These experiments indicate that in the WT enzyme and in the absence of copper, the NMBD interacts with the enzyme headpiece, interfering with the reverse formation of phosphoenzyme intermediate (i.e. aspartate) by utilization of P_i_.

**Figure 5:**
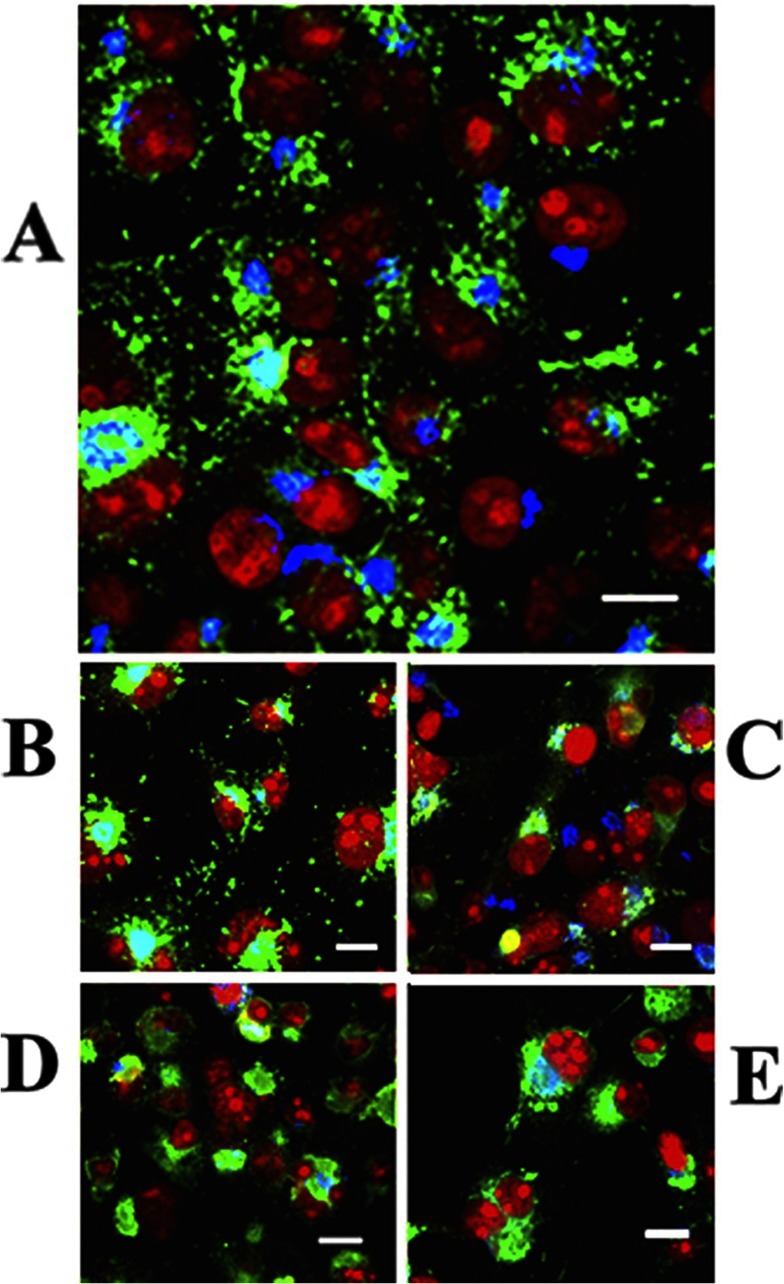
Intracellular distribution of ATP7B in COS-1 cells expressing WT ATP7B (A) or ATP7B subjected to mutations at Asp^1027^ (B), at Ser^478^, Ser^481^, Ser^1121^ and Ser^1453^ (C), at the transmembrane domain (TMBS) copper site (D) or at the sixth NMBD copper site (E) Note the presence of cytosolic trafficking vesicles of WT ATP7B and even following Asp^1027^ mutation, but no trafficking following serine, NMBD or TMBS copper site mutations. All panels present different fields of cells treated identically. A copper load (200 μM) was added to the culture medium 2 h following infection with adenovirus vector for delivery of ATP7B cDNA (WT or mutant). The cells were fixed 24 h later. Secondary antibodies were goat anti-mouse conjugated with Alexa Fluor® 488 for the ATP7B c-Myc tag (green) and donkey anti-rabbit conjugated with Cy5 (indodicarbocyanine) for Golgi (blue). Red indicates nuclei stained with propidium iodide. Scale bar, 10 μm. This Figure was adapted from J. Biol. Chem. [60] Pilankatta, R., Lewis, D. and Inesi G. Involvement of protein kinase D in expression and trafficking of ATP7B (copper ATPase). *Journal of Biological Chemistry*. 2011; **286**, 7389–7396.

The PKD involved in slow and alkaline-resistant phosphorylation is a serine/threonine kinase associated with the *trans*-Golgi network, regulating recruitment of transport carriers destined to the cell surface. In fact, studies on cultured cells sustaining heterologous expression show that nascent WT ATP7B transits to the Golgi complex [60] where it undergoes serine phosphorylation by PKD. It is of interest that serine phosphorylation protects nascent ATP7B from proteasome-mediated degradation. As a final step, phosphorylated ATP7B is transferred from the Golgi complex to cytosolic trafficking vesicles (Figure 5). This is observed even following Asp^1027^ mutation. On the other hand, phosphorylation and trafficking are completely prevented by mutations of NMBD (but not TMBS) copper-binding sites, demonstrating copper dependence of both PKD-assisted phosphorylation and trafficking. Furthermore, ATP7B trafficking is markedly reduced by Ser^478^/Ser^481^/Ser^1121^/Ser^1453^ to alanine mutations. It is therefore clear that PKD plays a key role in copper-dependent serine phosphorylation, permitting high levels of ATP7B protein expression and trafficking. PKD interaction with ATP7B is evidently dependent on the presence of the NMBD and its occupancy by copper.

It should be understood that the PKD-assisted phosphorylation of serine residues is implicated in cellular trafficking of mammalian copper ATPases, but is not required for the occurrence of the ATPase catalytic cycle itself. In fact, the catalytic cycle of bacterial copper ATPases occurs without any observable phosphorylation of serine residues.

**Figure 6:**
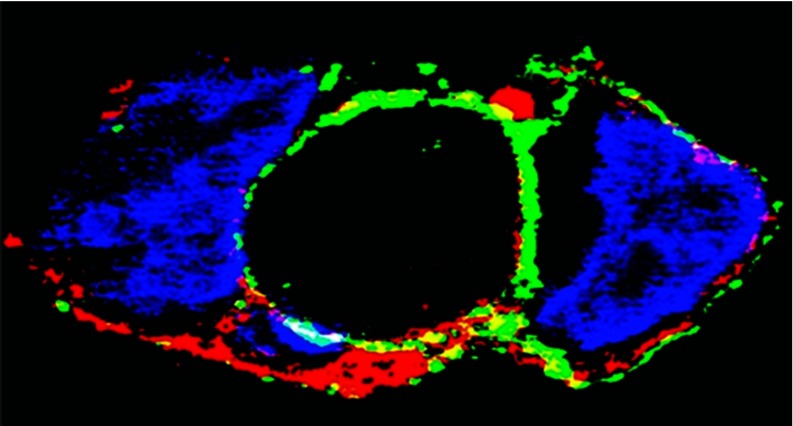
Association of ATP7A protein with the plasma membrane of COS-1 cells COS-1 cells were infected with optimal rAdATP7Amyc viral titres, and 200 μM copper was added. After 24 h, the cells were fixed with paraformaldehyde, permeabilized with Triton X-100, and stained. Immunostaining of ATP7A with the anti-Myc tag antibody is shown in green, and plasma membrane immunostaining with the anti-pan-cadherin antibody is shown in red. Nuclear staining with DAPI is shown in blue. Yellow indicates co-localization of ATP7A and anti-pan-cadherin antibodies in the plasma membrane. Scale bar, 10 μm. Reproduced from [61]. Copyright © 2010 American Chemical Society.

It is of interest that ATP7A, even though sustaining identical catalytic kinetics as ATP7B, has a lower level of serine phosphorylation, and a significant level of glycosylation which is not observed at all with ATP7B [61]. It is then apparent that a specific feature of ATP7A is glycosylation which, in conjunction with subtle changes of amino acid sequence [45], determines stabilization on plasma membranes (Figure 6), and may explain its functions in copper homoeostasis.

### Interactions with cisplatin

Cisplatin is a platinum-containing anticancer drug whose effectiveness is reduced following repeated administrations. Development of resistance is attributed to various mechanisms, including unfavourable drug accumulation and efflux by ATP7A and ATP7B, whose expression is often increased in parallel with cisplatin resistance [62–67]. Cisplatin is accumulated in membrane vesicles obtained from Sf9 cells expressing ATP7B, indicating active transport of the drug by the ATP7B ATPase [68]. On the other hand, expression of an ATP7B segment (NMBD) including four copper-binding motifs protects bacterial cells from toxic effects of cisplatin, suggesting that cisplatin binding to the ATPase NMBD is involved and may be sufficient to produce drug resistance [69,70].

**Figure 7:**
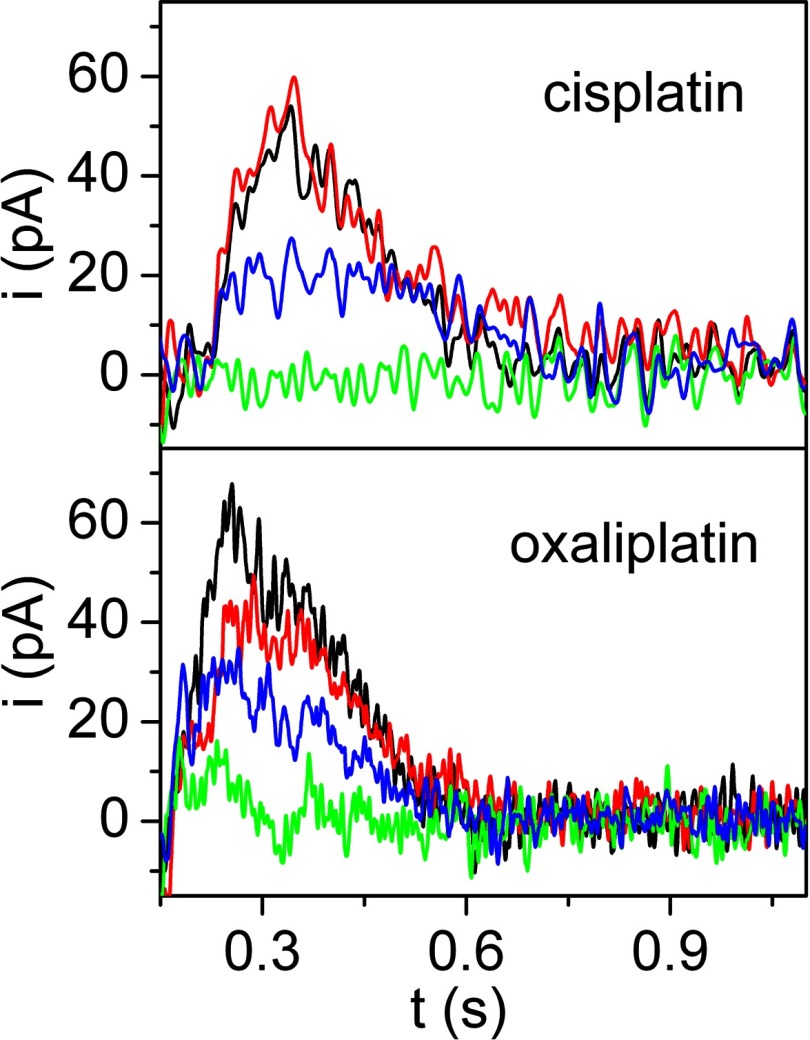
Current signals developed by ATP7A on a solid supported membrane in the presence of cisplatin or oxaliplatin Representative current transients induced by ATP (100 μM) concentration jumps in the presence of: CuCl_2_ (5 μM, black lines), cisplatin or oxaliplatin (5 μM, red lines), a mixture of CuCl_2_ and cisplatin or oxaliplatin (5 μM of each, green lines), and a mixture of CuCl_2_ and cisplatin or oxaliplatin (5 μM of each) with a copper chelator (1 mM BCS, blue lines). Adapted from [71]. Translocation of platinum anticancer drugs by human copper ATPases ATP7A and ATP7B. Tadini-Buoninsegni, F., Bartolommei, G., Moncelli, M. R., Inesi, G., Galliani, A., Sinisi, M., Losacco, M., Natile, G. and Arnesano, F. Angew. Chem. Int. Ed. Engl. **53**, Issue 5. Copyright © 2014 WILEY-VCH Verlag GmbH & Co. KGaA, Weinheim.

Recent electrical measurements performed with membrane fractions of COS-1 cells expressing ATP7A (or ATP7B), and adsorbed on to a solid supported membrane (see above for technical details), demonstrate the occurrence of charge transfer (Figure 7) upon addition of ATP in the presence of micromolar concentrations of cisplatin or oxaliplatin [71], in analogy with measurements performed in the presence of copper [58]. Furthermore, mutations within the NMBD segment, or of the Asp^1044^ residue involved in formation of phosphoenzyme intermediate, interfere with cisplatin-related charge transfer as well as with copper charge transfer. These experiments indicate that a charged cisplatin complex (presumably bearing the amine groups [71]) undergo ATP-dependent vectorial displacement by ATP7A and ATP7B with a mechanism analogous to that of copper, requiring occupancy of the NMBD sites for catalytic activation of the ATPase [58,72]. The cisplatin complex would then undergo transfer into cytosolic vesicles or across plasma membranes, which is likely to be a factor in the mechanism of cisplatin resistance in anticancer therapy.

In the absence of high-resolution structural information, it is difficult to predict how the cisplatin molecular mass may fit in the TMBS copper site. On the other hand, the presence of cisplatin in NMBD copper sites has been demonstrated directly by NMR spectroscopy [71]. Furthermore, it should be noted that simultaneous presence of copper and platinum does not yield displacement of either [71], suggesting that heterogeneous occupancy of copper sites by the two different metal ions interferes with the co-operative conformational change required for ATPase activation. On the other hand, if the concentration of free copper ion is lowered with a chelating agent, as presumably occurring *in vivo* due to binding to various specific proteins, cisplatin-related charge displacement is observed again [71].

## CONCLUSIONS

As described above, although PII-type ATPases sustain active transport of alkali and alkali-earth free ions (i.e. Na^+^, Ca^2+^) against electrochemical gradients across defined membranes, PIB-type ATPases undergo trafficking from/to various membrane compartments, transferring transition metal ions (i.e. Cu^+^) from delivery to acceptor proteins. In conformity with other P-ATPases, the free energy of ATP is utilized by copper ATPases through formation of a phosphoenzyme intermediate, yielding protein conformational changes that affect orientation and affinity of cation-binding sites. A specific feature of copper ATPases is the NMBD, which is required for catalytic regulation, and interaction with other proteins. Phosphorylation of serine residues and glycosylation are involved in interactions and trafficking of mammalian copper ATPases. Cisplatin, a platinum-containing anticancer agent, interacts with mammalian ATPase with a mechanism analogous to copper.

## Online data

Supplementary data
