# Supplementary material for: Biochemical characterization of P-type copper ATPases
Source: Biochem J. 2014 Sep 22;463(Pt 2):167–76. doi: 10.1042/BJ20140741 (PMC4179477; doi:10.1042/BJ20140741)
Supplement: Supplementary data [file bj4630167add.pdf]

BJ Biomolecules

Biochem. J. (2014) **463**, 167–176 (Printed in Great Britain) doi:10.1042/BJ20140741

SUPPLEMENTARY ONLINE DATA

Biochemical characterization of P-type copper ATPases

Giuseppe INESI*[1], Rajendra PILANKATTA† and Francesco TADINI-BUONINSEGNI‡

*California Pacific Medical Center Research Institute, San Francisco, CA 94920, U.S.A.
†Department of Biochemistry and Molecular Biology, Central University of Kerala, Padennakkad (P.O), Nileshwar, Kasaragod-671 314, Kerala, India
‡Department of Chemistry "Ugo Schiff", University of Florence, 50019 Sesto Fiorentino, Italy

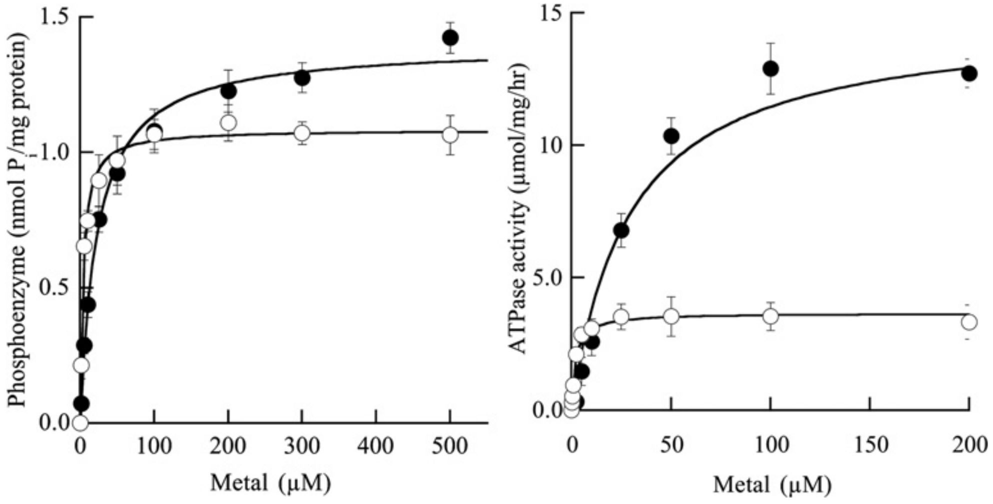

Figure S1 Ag^+ (●) and Cu^+ (○) dependence of phosphorylation of *A. fulgidus* CopA by ATP (left-hand panel) and ATPase activity (right-hand panel)

Enzyme phosphorylation with ATP was obtained at pH 7.5 for 30 s at 37°C. ATPase activity was assayed at pH 6.1 and 75°C, obtaining sequential samples for measurement of P_i. This Figure was originally published in J. Biol. Chem. [1] Mandal, A.K., Cheung, W.D. and Argüello, J.M. Characterization of a thermophilic P-type Ag^+/Cu^+-ATPase from the extremophile *Archaeoglobus fulgidus*. *Journal of Biological Chemistry*. 2002; **277**, 7201–7208. © the American Society for Biochemistry and Molecular Biology.

[1] To whom correspondence should be addressed (email ginesi@cpmcri.com).

© 2014 The Author(s)
This is an Open Access article distributed under the terms of the Creative Commons Attribution Licence (CC-BY) (http://creativecommons.org/licenses/by/3.0/) which permits unrestricted use, distribution and reproduction in any medium, provided the original work is properly cited.

Biochemical Journal www.biochemj.org

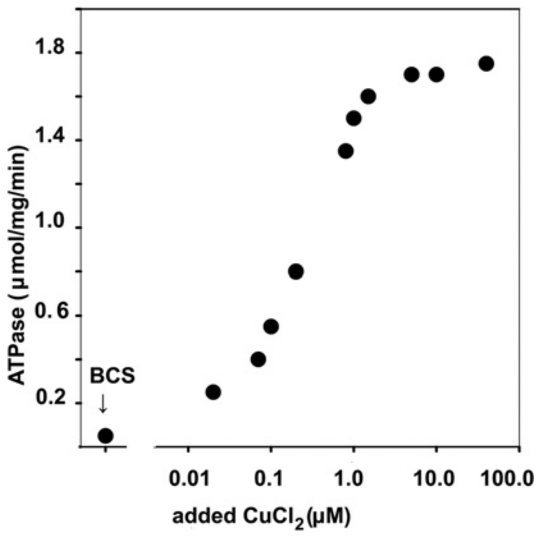

Figure S2 Copper dependence of steady-state ATPase activity of *T. maritima* CopA

ATPase activity was obtained at pH 6.0 and 60 °C, and measured by colorimetric determination of P_i. As indicated in the Figure, the measurements were obtained either in the presence of 1 mM BCS or in the absence of BCS following addition of incremental concentrations of $CuCl_2$. This Figure was originally published in J. Biol. Chem. [2] Hatori, Y., Hirata, A., Toyoshima, C., Lewis, D., Pilankatta, R. and Inesi, G. Intermediate phosphorylation reactions in the mechanism of ATP utilization by the copper ATPase (CopA) of *Thermotoga maritima*. *Journal of Biological Chemistry*. 2008; **283**, 22541–22549 © the American Society for Biochemistry and Molecular Biology.

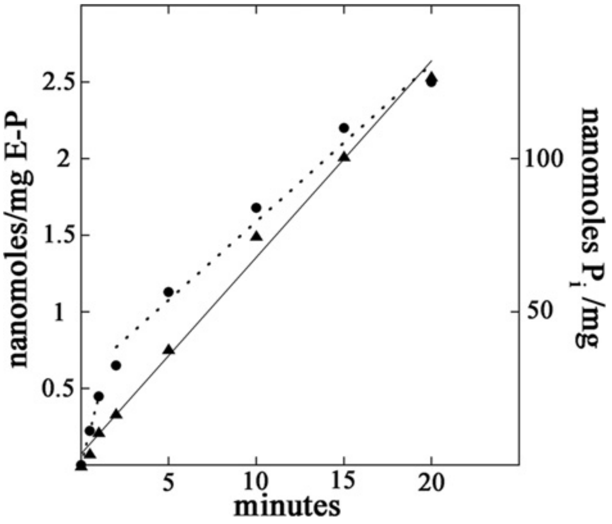

Figure S3 Formation of [γ-^{32}P]phosphoenzyme (●) and hydrolytic cleavage of [γ-^{32}P]P_i (▲) following addition of ATP to membrane fractions of COS1 cells sustaining heterologous expression of ATP7B

WT ATP7B was incubated with 50 μM [γ-^{32}P]ATP at 37 °C in a reaction mixture containing 50 mM Mes triethanolamine, pH 6.0, 300 mM KCl, 10 mM DTT, 3 μM $CuCl_2$ and 3 mM $MgCl_2$. Samples were quenched at serial times with 5 % trichloroacetic acid and filtered through 0.45-μm Millipore filters. The filtrate was removed and processed for determination of [^{32}P]P_i. The filters were then washed with 0.125 M ice-cold perchloric acid and ice-cold water, and used for determination of [γ^{32}P]phosphoenzyme. This Figure was adapted from J. Biol. Chem. [3] Pilankatta, R., Lewis, D., Adams, C.M. and Inesi, G. C. High yield heterologous expression of wild-type and mutant Cu^+-ATPase (ATP7B, Wilson disease protein) for functional characterization of catalytic activity and serine residues undergoing copper-dependent phosphorylation. *Journal of Biological Chemistry*. 2009; **284**, 21307–21316. © the American Society for Biochemistry and Molecular Biology.

© 2014 The Author(s)
This is an Open Access article distributed under the terms of the Creative Commons Attribution Licence (CC-BY) (http://creativecommons.org/licenses/by/3.0/) which permits unrestricted use, distribution and reproduction in any medium, provided the original work is properly cited.

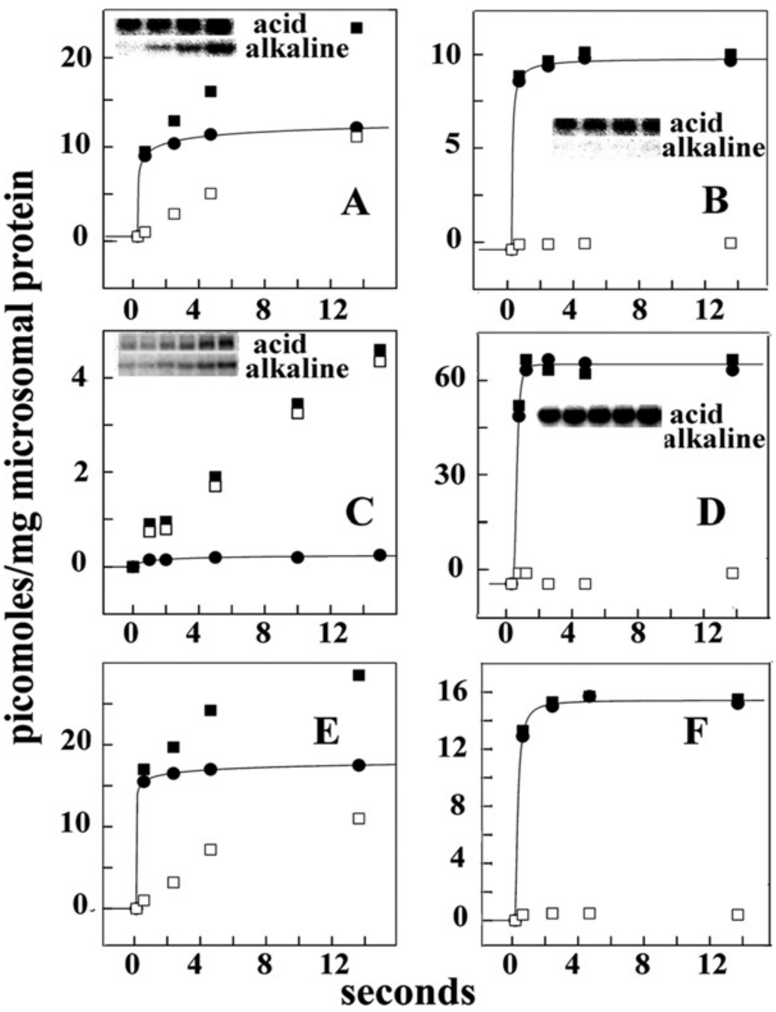

Figure S4 Phosphorylation of WT ATP7B (A, B, E and F), ATP7B D1027N mutant (C), and SERCA1 (D) in the absence (A, C, D and E) and presence (B and F) of PKD inhibitor

Note how the PKD inhibitor prevents formation of alkali-stable phoshoprotein in WT ATP7B (○), whereas the aspartate residue mutation (D1027N) prevents formation of the acid-stable phosphoenzyme intermediate (●). Alkali-stable phosphoenzyme was not formed in SERCA1 protein even in the absence of PKD inhibitor (**D**). Reactions performed with microsomes obtained from COS-1 cells (**A**, **B**, **C** and **D**) or HepG2 hepatocytes (**E** and **F**) sustaining heterologous expression of WT ATP7B (**A**, **B**, **E** and **F**), ATP7B D1027N mutant (**C**) or SERCA1 (**D**) were incubated with 50 μM [γ-^{32}P]ATP at 30°C in the absence (**A**, **C**, **D** and **E**) or presence (**B** and **F**) of 20 μM CID755673 (PKD inhibitor). Electrophoresis in acid buffer or alkaline buffer was then performed to distinguish total [^{32}P]phosphoprotein (■) from alkali-resistant [^{32}P]phosphoprotein (serine and/or threonine; □). The difference is considered alkali-labile [^{32}P]phosphoprotein (aspartate; **F**) and attributed to formation of phosphorylated enzyme intermediate. 'Acid' and 'alkaline' refer to the medium used for resuspension of samples and electrophoresis. The stoichiometry of phosphoprotein refers to total microsomal protein. (**A**) WT ATP7B from COS-1 cells. (**B**) WT ATP7B from COS-1 cells with 20 μM CID755673. (**C**) D1027N ATP7B from COS-1 cells. (**D**) SERCA1 from COS-1 cells. (**E**) WT ATP7B from HepG2 hepatocytes. (**F**) WT ATP7B from HepG2 hepatocytes with 20 μM CID755673. Note the different stoichiometric scale of the vertical axis in the left- and right-hand panels. Electrophoresis gel images correspond to sequential samples obtained within the time scale shown in the horizontal axis. This Figure was adapted from J. Biol. Chem [4] Pilankatta, R., Lewis, D. and Inesi G. Involvement of protein kinase D in expression and trafficking of ATP7B (copper ATPase). *Journal of Biological Chemistry*. 2011; **286**, 7389–7396. © the American Society for Biochemistry and Molecular Biology.

© 2014 The Author(s)

This is an Open Access article distributed under the terms of the Creative Commons Attribution Licence (CC-BY) (http://creativecommons.org/licenses/by/3.0/) which permits unrestricted use, distribution and reproduction in any medium, provided the original work is properly cited.

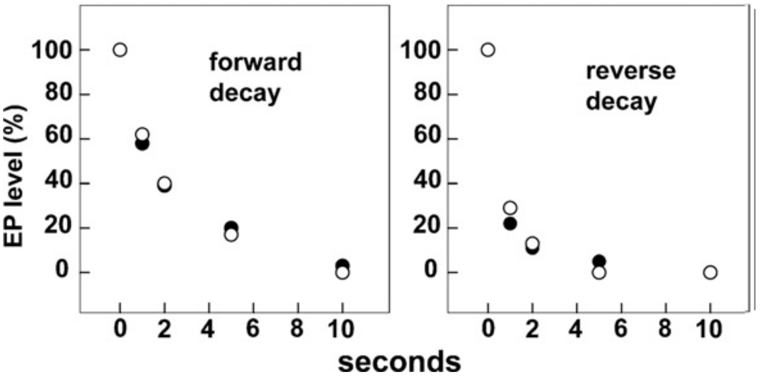

Figure S5 Decay of the [γ-^{32}P]phosphoenzyme intermediate obtained with [γ-^{32}P]ATP, in the forward and reverse direction of the catalytic cycle following addition of either non-radioactive ATP (forward) or ADP (reverse)

Microsomes obtained from COS-1 cells with sustained expression of ATP7A (black circles) or ATP7B (white circles) were reacted with [γ-^{32}P]ATP at 10°C for 15 s, at which time 1 mM non-radioactive ATP or ADP was added, and the samples were acid-quenched at various times as indicated. The quenched samples were dissolved in detergent at acidic or alkaline pH and analysed by electrophoresis at acidic or alkaline pH. The Figure displays residual acid-labile [^{32}P]phosphoprotein (i.e. phosphorylated enzyme intermediate) following addition of non-radioactive ATP (forward direction of the cycle) or ADP (reverse direction of the cycle). This Figure was adapted from J. Biol. Chem. [5] Lewis, D., Pilankatta, R., Inesi, G., Bartolommei, G., Moncelli, M.R. and Tadini-Buoninsegni, F. Distinctive features of catalytic and transport mechanisms in mammalian sarco-endoplasmic reticulum Ca^{2+} ATPase (SERCA) and Cu^{+} (ATP7A/B) ATPases. *Journal of Biological Chemistry* 2012; **287**, 32717–32727. © the American Society for Biochemistry and Molecular Biology.

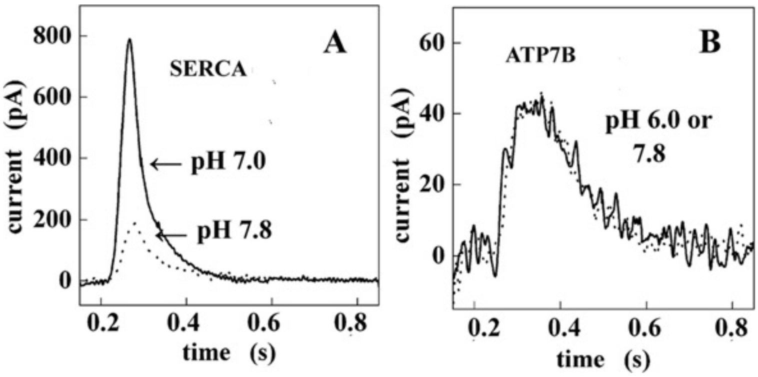

Figure S6 Charge measurements on native sarcoplasmic reticulum Ca^{2+} ATPase (SERCA) and recombinant Cu^{+} ATPase (ATP7B)

(**A**) Current transients were obtained after 100 μM ATP concentration jumps on SERCA in the presence of 10 μM free Ca^{2+} and 100 mM KCl at pH 7 (solid line) and 7.8 (dotted line). (**B**) Current transients obtained after 100 μM ATP concentration jumps on ATP7B in the presence of 5 μM $CuCl_2$ and 300 mM KCl at pH 6 (solid line) and 7.8 (dotted line). For each protein, the charge is normalized with respect to the value measured at pH 7 or pH 6.0. Note how alkaline pH reduces charge transfer in the Ca^{2+} ATPase, but makes no difference in the copper ATPase. The higher and lower currents in the two graphs reflect the different concentrations of ATPase in native and recombinant preparations. This Figure was adapted from J. Biol. Chem. [5] Lewis, D., Pilankatta, R., Inesi, G., Bartolommei, G., Moncelli, M.R. and Tadini-Buoninsegni, F. Distinctive features of catalytic and transport mechanisms in mammalian sarco-endoplasmic reticulum Ca^{2+} ATPase (SERCA) and Cu^{+} (ATP7A/B) ATPases. *Journal of Biological Chemistry* 2012; **287**, 32717–32727. © the American Society for Biochemistry and Molecular Biology.

© 2014 The Author(s)
This is an Open Access article distributed under the terms of the Creative Commons Attribution Licence (CC-BY) (http://creativecommons.org/licenses/by/3.0/) which permits unrestricted use, distribution and reproduction in any medium, provided the original work is properly cited.

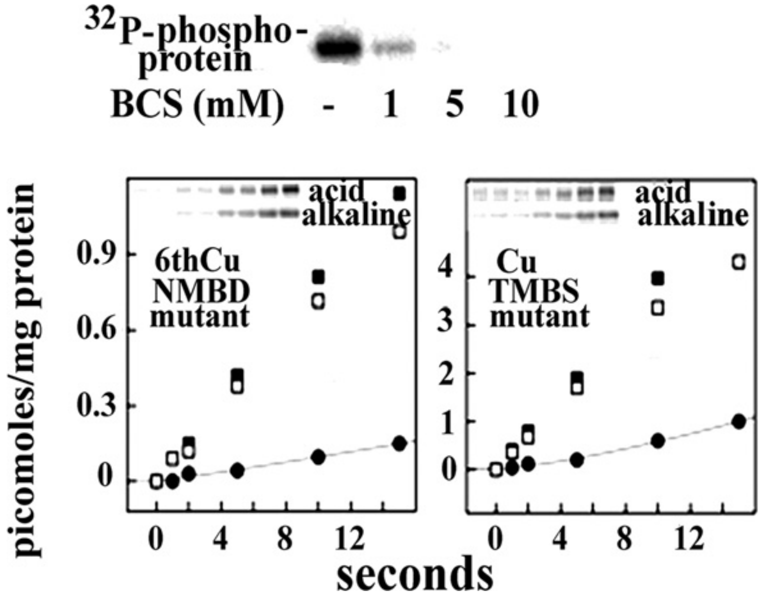

Figure S7 ATP7B phosphorylation following addition of [γ-^{32}P]ATP to microsomal fractions containing ATP7B: the copper NMBD site mutation inhibits both alkaline-resistant and acid-resistant phosphoenzyme formation; the copper TMBS mutation inhibits only the acid-stable phosphoenzyme intermediate formation

Following phosphorylation of microsomes obtained from COS-1 cells expressing ATP7B mutants, electrophoretic analysis was performed in acid or alkaline medium to detect total phosphoprotein (■) and distinguish alkali-labile (●; aspartyl phosphate) and alkali-stable (□; phosphorylated serine residues) fractions. 6th CuNMBD mutant: C575A/C578A mutation at the sixth NMBD copper site; CuTMBS mutant: C983A and C985A mutation of the TMBS. Note the different stoichiometric scale on the vertical axis of the two panels, indicating inhibition of aspartate phosphorylation by either mutation, but inhibition of serine phosphorylation only by the sixth NMBD copper site mutation. The gel above the graphs shows that no phosphorylation occurs if copper is chelated with millimolar BCS. This Figure was adapted from J. Biol. Chem. [4] Pilankatta, R., Lewis, D. and Inesi G. Involvement of protein kinase D in expression and trafficking of ATP7B (copper ATPase). *Journal of Biological Chemistry*. 2011; **286**, 7389–7396.

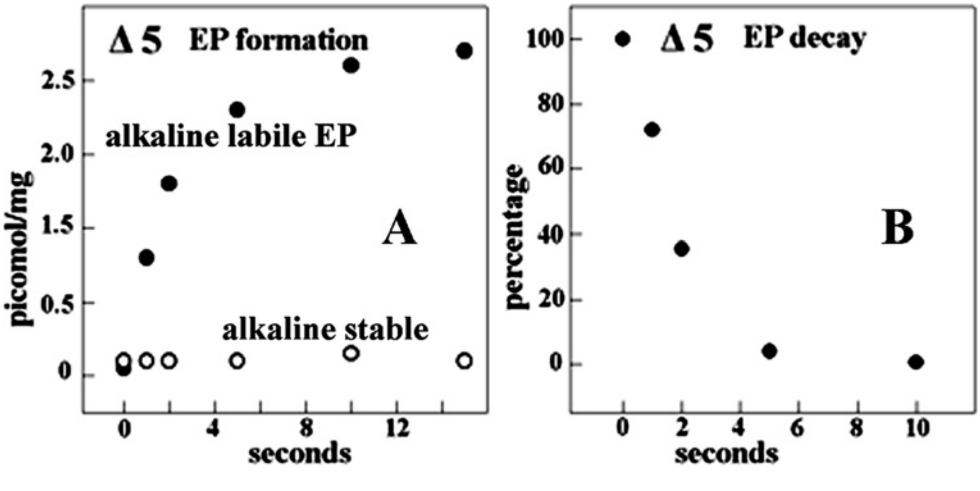

Figure S8 Functional behaviour of Δ5-ATP7B (NMBD segment with five out of six copper-binding sites deleted)

Formation of [γ-^{32}P]phosphoenzyme after [γ-^{32}P]ATP utilization by Δ5-ATP7B (**A**) and decay of [γ-^{32}P]phosphoenzyme after a chase with excess non-radioactive ATP (**B**). The experiments were performed at pH 6.0 and 10°C. Note that negligible (alkaline-stable) kinase-mediated serine/threonine phosphorylation is observed, due to NMBD deletion, whereas the phosphoenzyme intermediate is formed (**A**) and decays with normal kinetics (**B**). This Figure was adapted from J. Biol. Chem. [5] Lewis, D., Pilankatta, R., Inesi, G., Bartolommei, G., Moncelli, M.R. and Tadini-Buoninsegni, F. Distinctive features of catalytic and transport mechanisms in mammalian sarco-endoplasmic reticulum Ca^{2+} ATPase (SERCA) and Cu^{+} (ATP7A/B) ATPases. *Journal of Biological Chemistry* 2012; **287**, 32717–32727. © the American Society for Biochemistry and Molecular Biology.

© 2014 The Author(s)
This is an Open Access article distributed under the terms of the Creative Commons Attribution Licence (CC-BY) (http://creativecommons.org/licenses/by/3.0/) which permits unrestricted use, distribution and reproduction in any medium, provided the original work is properly cited.

REFERENCES

1 Mandal, A. K., Cheung, W. D. and Argüello, J. M. (2002) Characterization of a thermophilic P-type Ag^{+}/Cu^{+}-ATPase from the extremophile *Archaeoglobus fulgidus*. J. Biol. Chem. **277**, 7201–7208 CrossRef PubMed

2 Hatori, Y., Hirata, A., Toyoshima, C., Lewis, D., Pilankatta, R. and Inesi, G. (2008) Intermediate phosphorylation reactions in the mechanism of ATP utilization by the copper ATPase (CopA) of *Thermotoga maritima*. J. Biol. Chem. **283**, 22541–22549 CrossRef PubMed

3 Pilankatta,, R., Lewis, D., Adams, C. M. and Inesi, G. (2009) High yield heterologous expression of wild-type and mutant Cu^{+}-ATPase (ATP7B, Wilson disease protein) for functional characterization of catalytic activity and serine residues undergoing copper-dependent phosphorylation. J. Biol. Chem. **284**, 21307–21316 CrossRef PubMed

4 Pilankatta,, R., Lewis, D. and Inesi, G. (2011) Involvement of protein kinase D in expression and trafficking of ATP7B (copper ATPase). J. Biol. Chem. **286**, 7389–7396 CrossRef PubMed

5 Lewis, D., Pilankatta, R., Inesi, G., Bartolommei, G., Moncelli, M. R. and Tadini-Buoninsegni, F. (2012) Distinctive features of catalytic and transport mechanisms in mammalian sarco-endoplasmic reticulum Ca^{2+} ATPase (SERCA) and Cu^{+} (ATP7A/B) ATPases. J. Biol. Chem. **287**, 32717–32727 CrossRef PubMed

Received 11 June 2014/18 July 2014; accepted 6 August 2014
Published on the Internet 22 September 2014, doi:10.1042/BJ20140741

© 2014 The Author(s)
This is an Open Access article distributed under the terms of the Creative Commons Attribution Licence (CC-BY) (http://creativecommons.org/licenses/by/3.0/) which permits unrestricted use, distribution and reproduction in any medium, provided the original work is properly cited.
